# Circadian Blood Pressure Phenotyping Identifies Subtype-Specific Risk and Outcomes in Acute Ischemic Stroke: A Prospective Study

**DOI:** 10.1007/s12975-025-01400-x

**Published:** 2025-12-08

**Authors:** Priyanka Boettger, Jamschid Sedighi, Michael Buerke, Tobias Braun, Martin Juenemann, Omar Alhaj Omar

**Affiliations:** 1 Department of Cardiology, Angiology and Critical Care Medicine, Justus Liebig University, Klinikstrasse 33, 35392 Giessen, Hessen Germany; 2 Department of Neurology and Neurological Intensive Care, Justus Liebig University, Giessen, Hessen Germany; 3 Department of Cardiology, Angiology and Critical Care Medicine, St. Marien Hospital, Siegen, Nordrhein-Westphalia Germany

**Keywords:** Acute ischemic stroke, Blood pressure variability, Circadian rhythm, Blood pressure phenotypes, Early neurological deterioration, Functional outcome, Embolic stroke of undetermined source

## Abstract

Circadian organization of blood pressure (BP) is increasingly recognized as a determinant of vascular risk, but its role in acute ischemic stroke (AIS) remains undefined. We investigated whether early circadian BP phenotypes derived from high-frequency monitoring predict short- and long-term outcomes after AIS. In a prospective cohort of 529 patients with AIS, BP was recorded—noninvasively or via arterial line—during the first 72 h. Circadian parameters were extracted using cosinor analysis, and exploratory clustering was applied to identify recurrent BP patterns. Associations with early neurological deterioration (END), symptomatic intracerebral hemorrhage (sICH), 90-day functional outcome, mortality, and 1-year major adverse cardiovascular events (MACE) were assessed using multivariable logistic and Cox regression. Three distinct circadian BP phenotypes emerged: Steady-High (sustained elevation with blunted nocturnal decline), Disrupted-Rhythmicity (loss of amplitude and irregular oscillation), and Partial-Recovery (initial disorganization with progressive re-entrainment). Subtype-specific vulnerabilities were observed. In embolic stroke of undetermined source (ESUS), disrupted rhythmicity was associated with higher risk of END (adjusted OR 2.8, 95% CI 1.2–6.4). In cardioembolic stroke, circadian disorganization was linked to greater risk of sICH after reperfusion (adjusted OR 3.1, 95% CI 1.1–8.7). In large-artery atherosclerosis, the Steady-High phenotype predicted poor 90-day outcome (adjusted OR 2.2, 95% CI 1.0–4.6). Lacunar stroke showed relative preservation of circadian organization, with the lowest prevalence of adverse phenotypes. Across the cohort, Partial-Recovery was associated with the most favorable outcomes. High-frequency 72-hour BP monitoring combined with exploratory, data-driven clustering revealed reproducible circadian BP patterns with subtype-specific prognostic relevance in AIS. These findings suggest that circadian BP profiling may help inform individualized hemodynamic management in acute stroke.

**Supplementary Information:**

The online version contains supplementary material available at 10.1007/s12975-025-01400-x.

## Introduction

 Stroke remains one of the leading causes of death and long-term disability worldwide [1, 2], with early neurological deterioration and secondary complications critically shaping prognosis [3, 4]. Among vascular risk factors, hypertension is the single most important contributor to stroke incidence and recurrence, underscoring the central role of blood pressure across the continuum of cerebrovascular disease [5, 6]. In the acute phase, hemodynamic stability is a cornerstone of management because cerebral perfusion is highly dependent on systemic blood pressure [7]. Both sustained hypertension and excessive blood pressure lowering have been associated with adverse outcomes, highlighting the delicate balance required in the first days after stroke [8, 9].

Beyond absolute blood pressure levels, recent work has highlighted blood pressure variability (BPV) as an important determinant of outcome, reflecting impaired autoregulation and autonomic dysfunction [10]. Most studies, however, have assessed BPV using simple summary measures—such as standard deviation or coefficient of variation—over broad intervals [8, 11]. These approaches do not capture the temporal organization of blood pressure, particularly its circadian dynamics, which under normal physiology are characterized by a nocturnal decline and a daytime surge. Disruption of these rhythms has been linked to cardiovascular morbidity, yet their role in the immediate post-stroke setting remains poorly defined [12–14].

In addition, BPV is often considered a uniform construct, although patients may exhibit distinct early-phase phenotypes—such as stable, labile, or spiking trajectories—that could carry different prognostic implications. Clustering methods and data-driven phenotyping have been applied in hypertension and cardiovascular research [9, 15, 16], but systematic evaluation of such patterns in acute stroke is lacking. Addressing these gaps may provide new insights into cerebrovascular vulnerability and help refine individualized hemodynamic management. To date, very few studies have simultaneously addressed the circadian organization of blood pressure (BP) and the clustering of early BP phenotypes in acute stroke. Understanding how nocturnal rhythmicity and variability interact during the first 72 h may offer novel insights into cerebrovascular vulnerability after reperfusion and identify potential targets for individualized hemodynamic control.

We therefore aimed to (1) characterize circadian BP dynamics during the first three nights after acute ischemic stroke using high-resolution monitoring and cosinor modeling [17], (2) derive early-phase BP phenotypes through clustering of multiscale variability features, and (3) examine their associations with early neurological deterioration, symptomatic intracranial hemorrhage, and functional outcome at 90 days and 1 year. In addition, we explored potential effect modification by reperfusion status, stroke subtype, and patient characteristics such as sex and adiposity phenotype.

## Methods

We conducted a prospective observational cohort study of consecutive patients with acute ischemic stroke (AIS) admitted to the stroke unit. Patients were eligible if aged ≥ 18 years, admitted within 24 h of symptom onset, and had high-frequency blood pressure (BP) monitoring for ≥ 48 h. Stroke subtype was classified using TOAST criteria [18]; embolic stroke of undetermined source (ESUS) was defined according to international consensus [19] and analyzed as a predefined subgroup within the cryptogenic category; cases not fulfilling ESUS criteria were denoted as “cryptogenic (non-ESUS) for a better comparison. ESUS was defined as a non-lacunar infarct without ≥ 50% stenosis in the artery supplying the ischemic territory—stenosis that would otherwise denote large-artery atherosclerosis (LAA)—and without a major-risk cardioembolic source (e.g., atrial fibrillation, intracardiac thrombus, mechanical valve, recent myocardial infarction, or left ventricular dysfunction < 35%). Other specific etiologies such as arteritis or dissection were excluded. Lacunar stroke was defined as a single, small, subcortical infarct (≤ 20 mm on MRI or ≤ 15 mm on CT) within a perforator territory, lacking cortical involvement and evidence of large-artery disease.

### Exclusion Criteria

Patients with transient ischemic attack, primary intracerebral hemorrhage, early palliative care, or incomplete BP data were excluded. Additional exclusion criteria included sepsis, renal replacement therapy, end-stage kidney or liver disease, and continuous vasopressor therapy exceeding 3 h. Systemic comorbidities were assessed through admission laboratory data and clinical records (AST, ALT, bilirubin, and creatinine). Patients with acute hepatic failure, active malignancy, multiorgan failure, or endocrine disorders causing secondary hypertension (e.g., primary hyperaldosteronism or pheochromocytoma) were not included. To ensure physiologic validity of circadian recordings, datasets with inadequate monitoring density (< 12 h per night or > 20% missing values despite imputation), recurrent measurement artefacts (damped arterial waveform, cuff error, or limb contraindication), or persistent device malfunction were excluded.

### BP Acquisition and Analysis

Arterial blood pressure was captured either invasively (1-min averages) or non-invasively (scheduled every 15 min during the first 24 h and every 30–60 min thereafter). Invasive arterial monitoring was performed in 71 patients (9.9%), whereas 643 patients (90.1%) were monitored non-invasively on the stroke unit. Hypertension was defined according to current guidelines [20, 21]. Artefactual readings were removed a priori. Short gaps (≤ 30 min) were interpolated using Kalman smoothing [22]; longer gaps were left missing. The cohort-level median measurement density was 82% (IQR 75–88). Circadian dynamics over nights 1–3 were quantified by cosinor regression (MESOR, amplitude, acrophase) alongside stability/variability metrics. Detailed definitions, formulas, and units for all derived BP features are provided in Supplementary Table S8. Cosinor regression and all derived features (and clustering inputs) were estimated from *full 24-hour windows* (Cycle 1- Cycle 3) aligned to admission time. Night-time plots (21:00–09:00) are shown for interpretability because dipping is defined nocturnally and daytime readings are more confounded by activity and procedures.

### Dipping Status

was defined according to international ambulatory BP monitoring criteria (AHA/ESH) [23] as the percentage nocturnal decline in mean systolic BP relative to the preceding daytime mean:

$$\:\text{Nocturnal decline }(\%) = 100\times\frac{(\text{Daytime mean SBP}-\text{Nighttime mean SBP})}{\text{Daytime mean SBP}}.$$

Patients were categorized as “dippers” (≥ 10% and < 20% decline), “non-dippers” (< 10% decline), or “reverse-dippers” (nocturnal rise). Classification was based on cosinor-fitted mean BP values to reduce short-term variability.

### Lighting Environment and Circadian Conditions

All patients were treated in a stroke unit equipped with an automatically and spectrally tunable lighting system that reproduces natural circadian light–dark dynamics by continuously adjusting illuminance and color temperature (2,700–6,500 K) according to external luminance and solar altitude, and with a window façade that synchronizes indoor light spectra and intensity with outdoor luminance while minimizing glare. This environment provides a physiologically entrained circadian light exposure, justifying the use of the term circadian rather than diurnal throughout the manuscript.

### Risk Factors and Comorbidities

Vascular risk factors and Comorbidities were defined according to established clinical and imaging criteria. Body mass index (BMI) was calculated as weight in kilograms divided by height in meters squared; overweight and obesity were defined as BMI 25.0–29.9 kg/m² and ≥ 30 kg/m², respectively [24]. Hypertension was defined as a prior diagnosis, ongoing antihypertensive therapy, or office blood pressure ≥ 140/90 mm Hg [25, 26]. Diabetes mellitus was defined as a documented diagnosis, use of glucose-lowering medication, or fasting glucose ≥ 7.0 mmol/L or HbA1c ≥ 6.5% [27, 28]. Hypercholesterolemia was defined as a prior diagnosis, use of lipid-lowering therapy, or an LDL-cholesterol concentration ≥ 190 mg/dL (≥ 4.9 mmol/L) [29]. Atrial fibrillation (AF) was defined as a documented arrhythmia lasting >30 s on ECG or continuous cardiac monitoring, or as previously diagnosed AF [30]. Smoking status was classified as current, former, or never smoker; current smoking was defined as active tobacco use at admission or within the preceding 12 months [31]. Heart failure was defined according to ESC criteria as clinically established systolic dysfunction confirmed by echocardiography; in line with the ESUS framework, only HFrEF with LVEF < 35% was classified as heart failure in our study [32]. Coronary artery disease (CAD) was defined as a history of myocardial infarction or angiographically confirmed coronary atherosclerosis [33, 34]. A history of TIA or stroke was verified through medical records or neuroimaging; chronic infarcts were identified on MRI as focal lesions without restricted diffusion and on CT as well-demarcated areas of hypodensity consistent with prior infarction [35, 36]. Chronic kidney disease (CKD) was classified according to KDIGO 2024 criteria as eGFR < 60 mL/min/1.73 m² (G3a–G5) [37] based on glomerular filtration rate (GFR) only; proteinuria was not considered.

### Neuroimaging and Lesion Quantification

Baseline neuroimaging was performed according to institutional stroke protocols. MRI was conducted in 664 of 714 patients (93.0%) and included diffusion-weighted imaging (DWI) and MR angiography. Cranial CT was performed in 264 patients (37.0%), including CT angiography where indicated. Both imaging modalities were obtained in 104 patients (14.6%). Follow-up imaging was performed in 243 patients (34.0%). Lesions were segmented on DWI (preferred) or CT using semi-automated planimetric methods with manual correction by trained raters blinded to clinical data and BP phenotypes [38, 39]. Infarct volume was calculated by summing lesion areas across slices and expressed in milliliters [40, 41]. Vascular territory was categorized as anterior or posterior circulation based on arterial supply [42]. In patients treated with endovascular therapy, collateral grade was determined on multiphase CT angiography [43, 44], and reperfusion success was graded using the expanded Thrombolysis in Cerebral Infarction (eTICI) scale [45].

### Timing Parameters

Timing parameters included onset-to-door, door-to-needle, and, for patients undergoing EVT, puncture-to-reperfusion intervals. Symptom onset time (last known well) was prospectively recorded at admission—except in wake-up strokes or cognitively impaired patients—and categorized into 4-hour bins to assess circadian onset patterns. All timing variables were documented in the institutional stroke registry and entered as continuous covariates in multivariable models to adjust for treatment-related delays. Antihypertensive medication timing followed the fixed ward schedule at 06:00, 12:00, and 18:00 h. Additional intravenous bolus or infusion doses were time-stamped in the electronic medication administration record (eMAR). Indicators were created for scheduled daytime administration only versus any nighttime administration (22:00–05:59), and cumulative exposure during nights 1–3 was derived. Onset phase, medication timing, and cumulative exposure were included in prespecified sensitivity and interaction analyses.

### Phenotype Derivation

Multiscale BP features were standardized and entered into unsupervised clustering. The optimal number of clusters was determined through a stepwise evaluation of model performance using both geometric and information-based criteria. Specifically, candidate models with k ranging from 2 to 6 clusters were compared using the average silhouette width, which quantifies the internal cohesion and external separation of clusters (values approaching 1 indicate well-separated, compact clusters). In parallel, we assessed the Akaike Information Criterion (AIC) and Bayesian Information Criterion (BIC), which penalize model complexity to avoid overfitting. The combination of highest mean silhouette width and lowest AIC/BIC values consistently identified the three-cluster solution as optimal. To confirm robustness, we performed 1,000 bootstrap resamplings and found stable cluster membership probabilities (mean Jaccard similarity > 0.85 across resamples). Based on these complementary criteria, we retained three reproducible circadian BP phenotypes—Steady-High, Disrupted-Rhythmicity, and Partial-Recovery—for further analyses. This clustering approach was exploratory and data-driven, designed to reveal intrinsic BP trajectory patterns rather than impose predefined clinical categories.

### Outcomes

The primary outcome was early neurological deterioration (END, ≥ 4-point NIHSS increase within 72 h or death). Secondary outcomes were symptomatic intracerebral hemorrhage (sICH, Heidelberg definition [46, 47]), poor functional outcome at 90 days (mRS 3–6), mortality, and major adverse cardiovascular events (MACE) at one year.

### Synthetic Comparative Analysis

To contextualize the magnitude of circadian disruption, we performed a synthetic comparison using published data from large hypertensive non-stroke populations with 24-hour ambulatory blood pressure monitoring. Differences in the prevalence of non-/reverse-dipping and mean nocturnal systolic decline were assessed using two-sample Z-tests and t-tests, with standardized effect sizes expressed as Cohen’s *h*.

### Statistical Analysis

Baseline characteristics were summarized as mean ± SD, median (IQR), or frequencies and compared using χ² or ANOVA/Kruskal–Wallis tests, as appropriate. For categorical variables with expected cell counts < 5, Fisher’s exact test was applied to ensure statistical robustness. When overall group comparisons suggested potential heterogeneity, post hoc pairwise analyses were conducted using the Chi-square or Fisher’s exact test, applying Bonferroni correction for multiple comparisons when appropriate. Associations between circadian parameters or BP phenotypes and outcomes were estimated with multivariable logistic regression, adjusted for age, sex, baseline NIHSS, reperfusion therapy, glycemic status, temperature, antihypertensives, and infarct volume. Glycemic status was modeled comprehensively by including a diagnose of diabetes mellitus according to ADA [28] admission glucose, and HbA1c, thus accounting for both acute and chronic metabolic states and the presence of established disease. Results are presented as odds ratios (OR) with 95% confidence intervals (CI). Subgroup analyses assessed effect modification by reperfusion status, stroke subtype (including ESUS), sex, adiposity, and hypertension phenotype. Sensitivity and robustness analyses encompassed complete-case analysis, exclusion of arterial-line monitoring, high-density monitoring subsets, multiple imputation procedures, alternative variability metrics, and model specification checks. Detailed results are provided in Supplementary Tables S1–S7. Circadian dynamics over nights 1–3 were quantified by cosinor regression (MESOR, amplitude, acrophase) alongside stability and variability metrics. Extended sensitivity analyses incorporated infarct volume, vascular territory, onset-to-treatment intervals, reperfusion therapy (IVT, final eTICI), collateral grade, and etiologic subtype (TOAST/ESUS classification). Model calibration was assessed using the Hosmer–Lemeshow goodness-of-fit test, and multicollinearity was evaluated using variance inflation factors (VIF < 2.0 for all covariates). Missing data for imaging and metabolic covariates were handled by multiple imputation using chained equations (m = 20) under the missing-at-random assumption [48] Interaction terms (phenotype × sex, phenotype × treatment) were evaluated using false-discovery-rate correction [49]. Results of these extended models are presented in Supplementary Table S9. Analyses were performed in R (version 4.3.2). A two-sided *p* < 0.05 was considered statistically significant.

## Results

### Cohort and Baseline

**Fig. 1:**
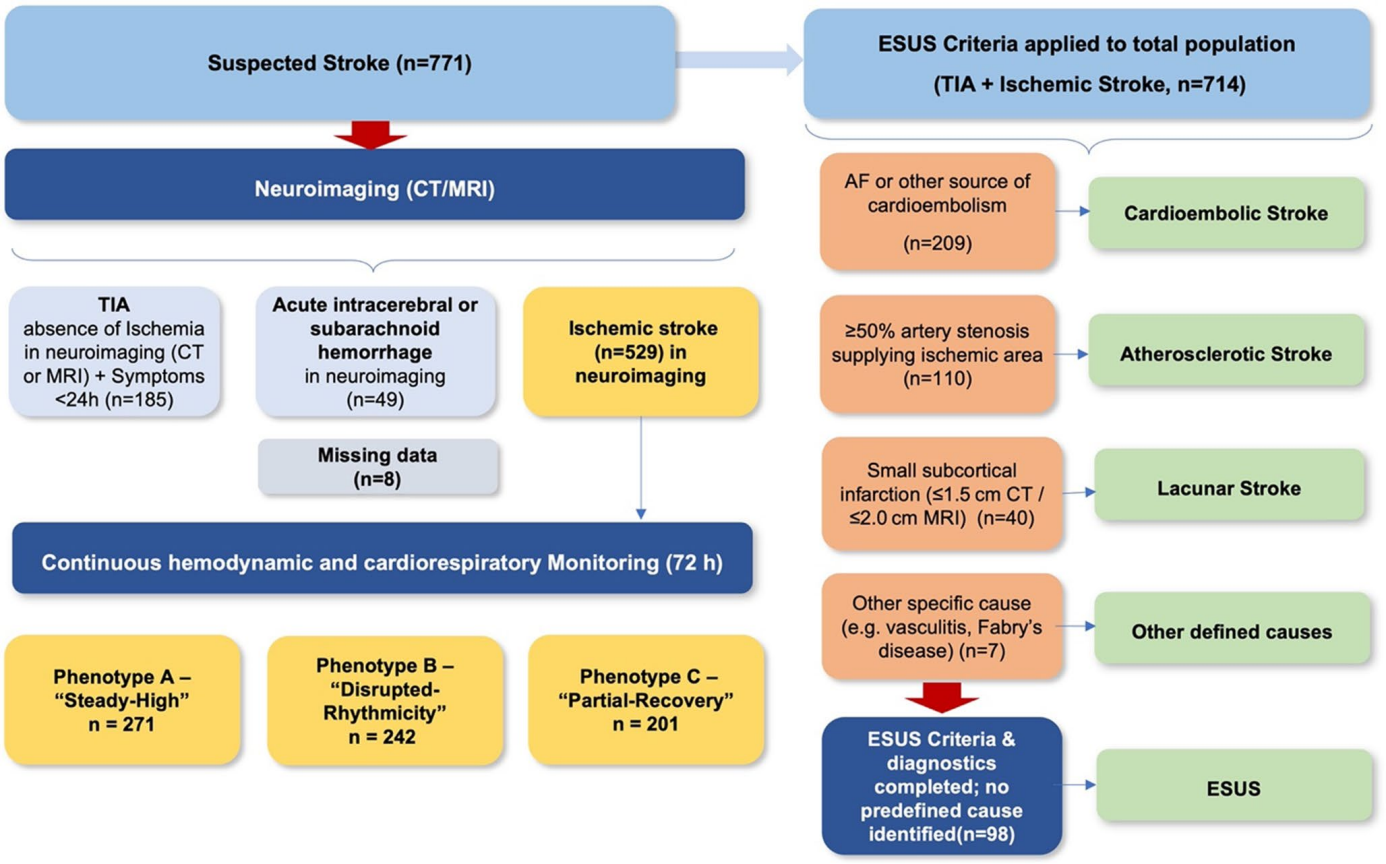
Study population and stroke subtype classification. From an initial cohort of 714 patients with acute ischemic stroke or TIA, 185 patients with transient ischemic attack (symptoms <24 hours and no ischemic lesion on imaging) were excluded. Of the remaining 529 patients, etiological classification identified cardioembolic stroke (n=209), atherosclerotic stroke (n=110), and lacunar stroke (n=40). Seven patients were assigned to other defined causes. Among 163 patients with cryptogenic stroke, 98 fulfilled the criteria for embolic stroke of undetermined source (ESUS), while 65 did not meet ESUS criteria

A total of 714 patients with acute ischemic stroke or TIA were included (mean age, 71 years; 311 [43.5%] women) (Fig. 1). In our cohort, most strokes occurred in the early morning hours between 5:00 and 10:00 AM. Among patients with known symptom onset (*n* = 430), 266 (62.0%) experienced stroke onset during this interval, coinciding with the physiological morning blood pressure surge, whereas 99 (23.0%) occurred at night (10:00 PM–4:59 AM) and 65 (15.0%) during the late afternoon or evening. Baseline characteristics by stroke subtype are shown in Table 1. Patients with cardioembolic stroke were older (median age, 77 years) than those with ESUS (70 years) or lacunar stroke (72 years; *p* < 0.001). The study population comprised adults aged 20 to 99 years (mean age 73 ± 9 years), all of whom underwent continuous ECG monitoring for at least 24 h (mean 37 ± 7 h, range 24–55 h) using stroke-unit telemetry or Holter ECG. Women were most frequent in TIA (55.1%) and least in atherosclerotic stroke (32.7%; *p* = 0.01).

**Table 1:** Baseline characteristics by stroke subtype

Characteristic	All strokes(*n* = 714)	TIA(*n* = 185)	Cryptogenic (*Including ESUS; n* = 163)	ESUS(*n* = 98)	Athero sclerotic (*n* = 110)	Cardio embolic(*n* = 209)	Lacunar (*n* = 40)	*P* value
Female sex	311 (43.5)	102 (55.1)	61 (37.4)	38 (38.8)	36 (32.7)	90 (43.1)	18 (45.0)	0.002
Age, years (mean ± SD)	71 ± 13	72 ± 12	67 ± 14	67 ± 13	70 ± 13	75 ± 11	72 ± 11	—
Previous TIA or stroke	181 (25.4)	41 (22.2)	37 (22.7)	11 (11.2)	36 (32.7)	55 (26.3)	10 (25.0)	0.012
Nicotine abuse	198 (27.7)	44 (23.8)	48 (29.4)	28 (29.0)	45 (41.0)	51 (25.0)	7 (17.5)	0.013
Obesity (BMI ≥ 30 kg/m²)	334 (46.7)	75 (40.5)	84 (51.5)	53 (54.2)	45 (41.0)	106 (50.7)	19 (47.5)	0.103
Hypertension	537 (75.2)	151 (81.6)	116 (71.0)	71 (72.2)	83 (75.5)	156 (74.6)	29 (72.5)	0.295
Diabetes mellitus	212 (29.7)	43 (23.0)	47 (28.8)	31 (31.6)	39 (35.5)	66 (31.6)	16 (40.0)	0.159
Atrial fibrillation	163 (22.8)	42 (22.7)	0 (0.0)	0 (0.0)	14 (12.7)	101 (48.3)	5 (12.5)	< 0.001
Heart failure	66 (9.0)	6 (2.7)	14 (7.3)	6 (6.1)	5 (4.5)	40 (19.3)	1 (2.5)	< 0.001
Hypercholesterolemia	274 (38.4)	68 (36.8)	66 (40.5)	42 (42.9)	59 (53.6)	66 (31.6)	16 (40.0)	0.007
Admission ECG available	700 (98.0)	180 (97.3)	159 (97.5)	96 (98.0)	107 (97.3)	201 (96.2)	40 (100.0)	0.808
Stroke unit monitoring	651 (91.2)	165 (89.2)	151 (92.6)	94 (96.0)	101 (91.8)	191 (91.4)	36 (90.0)	0.524
Stroke unit monitoring time, h (mean ± SD)	37.1 ± 9.3	30.6 ± 7.7	37.9 ± 9.5	36.0 ± 9.0	39.9 ± 10.0	42.5 ± 10.6	34.1 ± 8.5	—

Continuous variables are expressed as mean ± SD, and categorical variables as n (%). P-values were derived from χ² or ANOVA/Kruskal–Wallis tests, with Fisher’s exact test applied for categorical variables with expected counts < 5. Total n per subgroup are shown in the column headers.The ‘Cryptogenic (n = 163)’ column reflects the entire cryptogenic group (non-ESUS cryptogenic stroke n = 65 and ESUS n = 98). ESUS is displayed as a separate column to allow subtype-specific comparison. Global P-values were calculated across TOAST subtypes and ESUS separately. Abbreviations: *TIA* transient ischemic attack; *ESUS* embolic stroke of undetermined source; *BMI *body mass index; *ECG* electrocardiogram;

Hypertension was the most common risk factor (75.2%) across subtypes. Diabetes was more common in lacunar stroke (40.0% vs. 29.7%; adjusted *p* = 0.04), smoking in large-artery atherosclerosis (41.0%; *p* < 0.001), and obesity in ESUS (54.2%; *p* = 0.03). Post hoc χ² tests with Bonferroni correction were applied where overall associations were significant. Atrial fibrillation was present in 48.3% of cardioembolic strokes but absent in ESUS (*p* < 0.001). Admission stroke severity varied, highest in cardioembolic strokes (mean NIHSS 11.0) and lowest in lacunar strokes (4.5; *p* < 0.001). Overall in-hospital mortality was 5.0%, highest in cardioembolic stroke (10.0%), and absent in ESUS and TIA (*p* = 0.02). Antihypertensive exposure is summarized in Table S7: approximately two-thirds of patients were on antihypertensive therapy before the index event, and during nights 1–3 more than 70% received at least one agent (ACEi/ARB, beta-blocker, or calcium-channel blocker most common; intravenous bolus/infusion use in roughly 15–20%). Exposure patterns were broadly similar across phenotypes and subtypes and, after covariate adjustment, did not materially alter the phenotype–outcome associations (see Table S5).

### Circadian Blood Pressure Dynamics in Nights 1–3

**Fig. 2:**
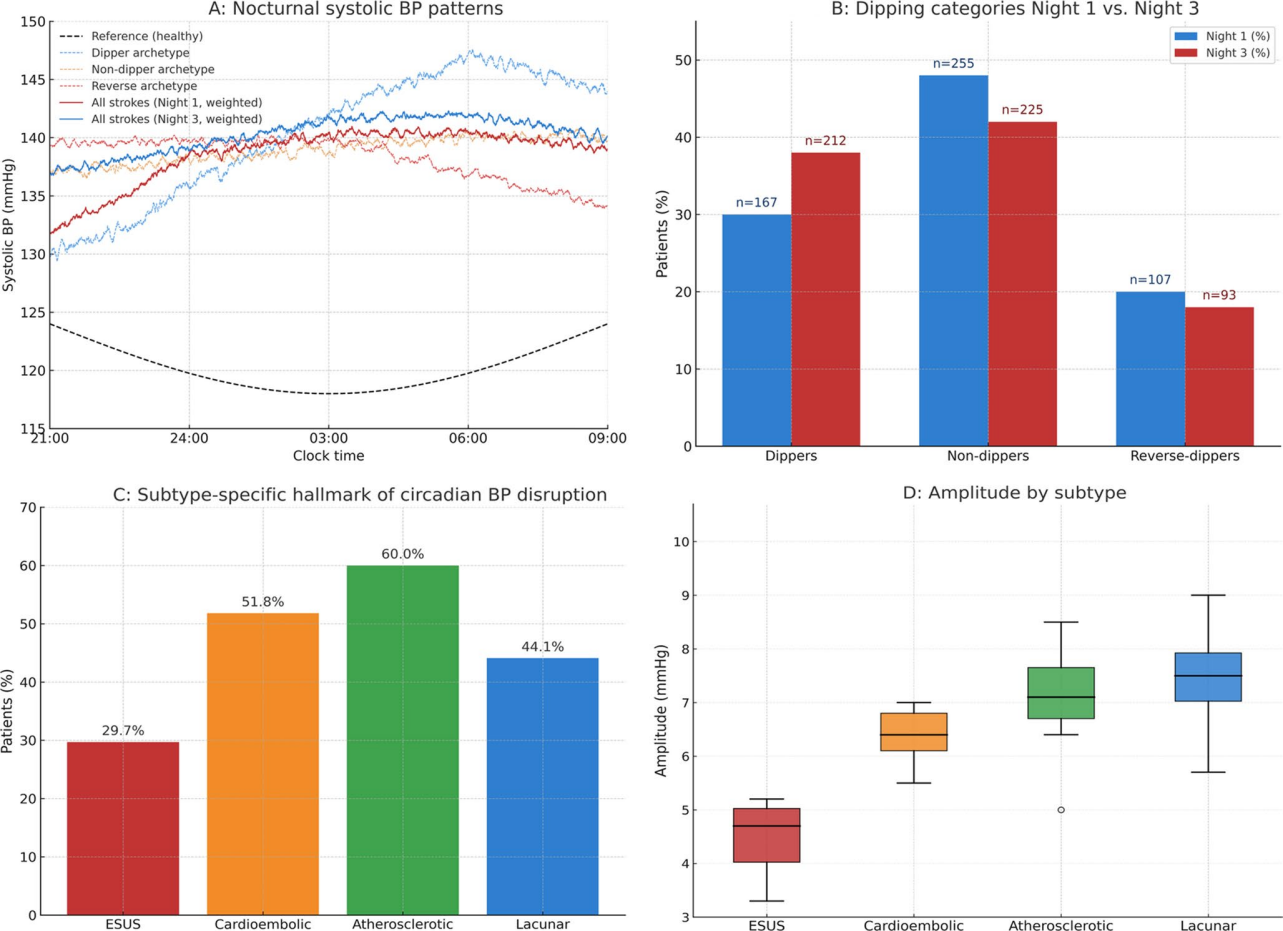
Circadian blood pressure dynamics during the first 72 hours after acute ischemic stroke. Panels **A**–**C** display nocturnal (21:00–09:00) segments to visualize dipping/non-dipping; models were fitted on full 24-hour data. The corresponding 24-hour trajectories are shown in Supplementary Figure S1.Panel** A:** Cohort-weighted nocturnal systolic blood pressure curves (21:00–09:00) for night 1 and night 3, compared with archetypal dipping, non-dipping, and reverse-dipping profiles. A blunted nocturnal decline with a delayed acrophase is evident in the stroke cohort. Panel** B:** Distribution of dipping categories, with a low prevalence of dippers on night 1 (31.5%) and only partial recovery by night 3 (40.0%), while non-dipping and reverse-dipping patterns remained overrepresented compared with population norms. Absolute n values are displayed to facilitate subgroup interpretation (ESUS n = 98; cardioembolic n = 209; atherosclerotic n = 110; lacunar n = 40). Panel** C**: Subtype-specific hallmarks of circadian blood pressure disruption. Bars illustrate distinct rhythmicity indices—such as prevalence of dipping categories and mean nocturnal pressure (MESOR)—on a shared percentage axis for visual consistency. Values reflect within-metric distributions and are not intended for direct comparison across metrics. Reverse-dipping was most frequent in ESUS (29.7%), non-dipping predominated in cardioembolic stroke (51.8%), persistently elevated nocturnal pressure was typical for atherosclerotic stroke (MESOR ≥ 140 mmHg in 60.0%), and dipping remained comparatively preserved in lacunar stroke (44.1%). Panel** D**: Amplitude by stroke subtype, with lowest values in ESUS (mean 4.9 mmHg), consistent with the most pronounced loss of circadian rhythmicity

Cosinor analyses revealed a marked disruption of circadian blood pressure organization in the first 72 h after stroke. Compared with reference values, amplitude was significantly blunted across all patients (mean 6.4 mmHg, 95% CI 5.2–7.6; *p* < 0.001), and MESOR remained elevated throughout nights 1–3 (overall mean 137 mmHg, 95% CI 135–139; *p* < 0.01 vs. normative data). Acrophase was delayed by 1.6 h (observed 06:30 vs. expected 04:45; *p* = 0.004). While Fig. 2 focuses on nocturnal segments for interpretability, 24-hour cohort-level trajectories for Cycle 1 and Cycle 3 and the corresponding daytime segment (09:00–21:00) are provided in Supplementary Figure S1. Dipping patterns were profoundly altered. On night 1, 167 (31.5%) patients were classified as dippers, 256 (48.2%) as non-dippers, and 108 (20.3%) as reverse-dippers, differing significantly from population norms (*p* < 0.001) (Fig. 2). By night 3, partial recovery was observed, with 212 (40.0%) dippers, while non-dipping (226 [42.6%]) and reverse-dipping (93 [17.5%]) remained markedly prevalent (*p* < 0.01). Subtype analyses revealed distinct circadian profiles. ESUS patients exhibited the greatest amplitude loss (mean 4.9 mm Hg; 95% CI, 3.6–6.2; *p* = 0.002 vs. other subtypes) and the highest prevalence of reverse-dipping (44 [29.7%]). Cardioembolic strokes showed a predominance of non-dipping (86 [51.8%]), whereas large-artery atherosclerosis was associated with persistently elevated nocturnal MESOR (steady-high profile; mean 148 mm Hg; *p* = 0.01 vs. lacunar). Patients with lacunar stroke displayed comparatively preserved rhythmicity, with the largest proportion of dippers (52 [44.1%]).

### Data-Driven Early Blood Pressure Phenotypes

**Fig. 3:**
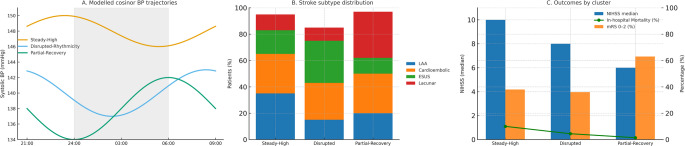
Data-driven early blood pressure phenotypes after acute ischemic stroke. Panel **A**: Modelled circadian blood pressure trajectories over the first 72 hours derived from cosinor analysis, stratified into three clusters: Steady-High (blue), Disrupted-Rhythmicity (orange), and Partial-Recovery (green). Shaded area indicates nighttime (24:00–06:00). Panel **B**: Stroke subtype distribution within each phenotype, displayed as stacked bar charts. Large-artery atherosclerosis (blue), cardioembolic (orange), ESUS (green), and lacunar (red) strokes show differential clustering patterns. Panel **C**: Clinical outcomes by phenotype. Blue bars indicate median NIHSS at admission, orange bars represent the proportion of patients with favorable outcome at 90 days (mRS 0–2), and the green line denotes in-hospital mortality. Patients in the Partial-Recovery cluster showed lower baseline severity and more favorable outcomes compared to the Steady-High and Disrupted-Rhythmicity groups

Unsupervised clustering of circadian BP trajectories in the first 72 h identified three distinct early BP phenotypes (Fig. 3). The optimal model was defined by Bayesian Information Criterion (BIC), supporting a 3-cluster solution.

- **Phenotype A (“Steady-High”)**: characterized by persistently elevated nocturnal systolic BP (MESOR = 148 mmHg), blunted amplitude (mean 5.1 mmHg), and low dipping prevalence (non-dippers 61.3%). This cluster comprised 38% of patients and was enriched in large-artery atherosclerosis.
- **Phenotype B (“Disrupted-Rhythmicity”)**: defined by markedly reduced amplitude (4.3 mmHg) and the highest prevalence of reverse-dipping (26.8%). This pattern was observed in 34% of patients, with overrepresentation of ESUS.
- **Phenotype C (“Partial-Recovery”)**: showed relatively preserved dipping (dippers 45.6%) and intermediate MESOR (134 mmHg), with gradual improvement from night 1 to night 3. This group comprised 28% of patients, enriched in lacunar strokes.

Clinical characteristics differed significantly across phenotypes (Table 2). Patients in the Steady-High cluster were older (median 76 years) and more hypertensive, while those in the Disrupted-Rhythmicity cluster had fewer traditional vascular risk factors but higher rates of cryptogenic stroke. The Partial-Recovery cluster showed the lowest baseline NIHSS and the most favorable functional outcome at 90 days (mRS 0–2: 63.5%, *p* < 0.01).

**Table 2:** Baseline and clinical characteristics according to early blood pressure phenotypes. Three distinct BP phenotypes were identified by unsupervised clustering of 72-hour circadian BP trajectories. The *Steady-High* phenotype was characterized by older age, higher prevalence of hypertension, enrichment of large-artery atherosclerosis, and the highest in-hospital mortality. The *Disrupted-Rhythmicity* phenotype showed fewer conventional vascular risk factors but was enriched in ESUS and had intermediate outcomes. The *Partial-Recovery* phenotype was enriched in lacunar stroke, displayed the lowest baseline NIHSS, and was associated with the most favorable functional outcome at 90 days

Characteristic	Phenotype A (“Steady-High”) *n* = 201 (38.0)	Phenotype B (“Disrupted-Rhythmicity”) *n* = 180 (34.0)	Phenotype C (“Partial-Recovery”) *n* = 148 (28.0)	*P* value
Demographics				
Age, years, median [IQR]	76 [69–82]	71 [63–79]	68 [60–76]	< 0.001
Female sex	86 (42.8)	79 (43.9)	64 (43.2)	0.97
Vascular risk factors				
Hypertension	168 (83.6)	128 (71.1)	103 (69.6)	0.002
Diabetes mellitus	74 (36.8)	51 (28.3)	37 (25.0)	0.04
Smoking	42 (20.9)	59 (32.8)	31 (20.9)	0.03
Stroke subtype				
Large-artery atherosclerosis	72 (35.8)	21 (11.7)	17 (11.5)	< 0.001
Cardioembolic	61 (30.3)	57 (31.7)	51 (34.5)	0.68
ESUS	32 (15.9)	54 (30.0)	12 (8.1)	< 0.001
Lacunar	18 (9.0)	16 (8.9)	47 (31.8)	< 0.001
Stroke severity and outcomes				
Baseline NIHSS, median [IQR]	10 [6–17]	8 [5–15]	6 [4–11]	< 0.001
90-day mRS 0–2	71 (35.3)	61 (33.9)	94 (63.5)	< 0.001
In-hospital mortality	17 (8.5)	8 (4.4)	2 (1.4)	0.01

Values are n (%) unless otherwise indicated. P values indicate global group differences based on χ² or Kruskal–Wallis tests, as appropriate. Post hoc pairwise χ² tests with Bonferroni correction were applied for significant overall results. Abbreviations: *ESUS *embolic stroke of undetermined source; *NIHSS* National Institutes of Health Stroke Scale; *mRS *modified Rankin Scale.

### Time-of-Day of Stroke Onset and Medication Timing

Among patients with known symptom onset (*n* = 430), 266 (62%) occurred between 05:00 and 10:00, 99 (23%) at night (22:00–04:59), and 65 (15%) in the late afternoon or evening. Morning onset was associated with a higher early prevalence of non-dipping and reverse-dipping profiles (global χ², *p* < 0.01), yet inclusion of onset phase as a covariate did not materially alter phenotype–outcome associations (all ΔaOR < 0.10). Regarding antihypertensive therapy, 73–76% of patients received exclusively the scheduled daytime doses (06:00/12:00/18:00), while 24–27% received at least one nighttime dose (22:00–05:59)—mainly short-acting intravenous agents. Modeling day- vs. night-time administration and cumulative exposure during nights 1–3 did not change the results (all ΔaOR < 0.10), and no significant phenotype × timing interactions were observed after false-discovery-rate correction. Details are provided in Supplementary Table S7.

### Clinical Outcomes at 72 Hours, 90 Days, and 1 Year

#### Early Outcomes by Etiologic Subtype

Early outcomes differed significantly across stroke subtypes. Cardioembolic stroke presented with the highest baseline severity (NIHSS, 11 [6–18]) compared with **ESUS ** (6 [3–10]), large-artery atherosclerosis (7 [4–12]), and small-vessel disease (6 [3–9]; *p* < 0.001). At discharge, NIHSS remained higher in cardioembolic stroke (8 [4–15]) than in ESUS (5 [2–9]), large-artery atherosclerosis (6 [3–10]), or small-vessel disease (5 [2–8]; *p* < 0.001). Functional disability was greater in cardioembolic stroke (mRS, 3 [2–5]) than in ESUS (2 [1–3]), large-artery atherosclerosis (3 [2–4]), or small-vessel disease (2 [1–3]; *p* < 0.001). In-hospital mortality was 20 (10.0%) for cardioembolic stroke, 9 (5.3%) for large-artery atherosclerosis, 4 (2.7%) for small-vessel disease, and 0 (0%) for ESUS. Circadian blood-pressure phenotypes provided additional prognostic information beyond stroke subtype (Table 3).

**Table 3:** Associations of early BP phenotypes with acute and long-term outcomes

Outcome	Steady-High (*n* = 201)	Disrupted-Rhythmicity (*n* = 180)	Partial-Recovery (*n* = 148)	*P* value
END ≤ 72 h	43 (21.5) | aOR 1.88 (95% CI 0.90–3.96)	51 (28.4) | aOR 2.57 (1.08–6.10)	19 (12.6)	0.008
sICH	18 (9.1) | aOR 2.86 (1.00–8.25)	8 (4.7) | aOR 1.37 (0.52–3.58)	3 (2.1)	0.030
mRS 0–2 at 90 d	83 (41.2) | aOR 0.50 (0.29–0.86)	82 (45.8) | aOR 0.59 (0.34–1.03)	94 (63.5)	< 0.01
Mortality (in-hospital/90 d)	20 (10.0) | aOR 3.18 (1.02–9.30)	8 (4.4) | aOR 1.36 (0.46–4.06)	2 (1.4)	0.040

Values are* n* *(%) *unless otherwise indicated. Adjusted odds ratios (aOR) were derived from multivariable logistic regression models adjusted for age, sex (except in sex-stratified analyses), baseline NIHSS, hypertension, glycemic status (diabetes, admission glucose, and HbA1c), atrial fibrillation, and treatment modality (IVT and/or EVT as applicable). Interaction terms tested the phenotype × modifier effects within multivariable logistic regression models. Reference category: Partial-Recovery phenotype. Abbreviations: *END* early neurological deterioration; *sICH* symptomatic intracerebral hemorrhage; *mRS *modified Rankin Scale; *EVT* endovascular therapy; *IVT* intravenous thrombolysis.

Early neurological deterioration occurred in 51 (28.3%) of Disrupted-Rhythmicity patients compared with 19 (12.8%) in Partial-Recovery (aOR 2.57; 95% CI 1.08–6.10; *p* = 0.03). The Steady-High phenotype conferred the greatest risk of symptomatic intracerebral hemorrhage after reperfusion, with 18 (9.0%) vs. 3 (2.0%) in Partial-Recovery (aOR 2.86; 95% CI 1.00–8.25; *p* = 0.03), and showed higher in-hospital mortality within BP phenotypes (20 [10.0%] vs. 2 [1.4%]; *p* = 0.04).

#### 90-Day Outcomes

Functional independence (mRS 0–2) was achieved in 94 (63.5%) Partial-Recovery, 82 (45.6%) Disrupted-Rhythmicity, and 83 (41.3%) Steady-High (*p < 0.01*). In adjusted models, Steady-High (aOR 0.50; 95% CI 0.29–0.86) and Disrupted-Rhythmicity (aOR 0.59; 95% CI 0.34–1.03) were independently associated with lower odds of functional independence. Ninety-day mortality was 20 (10.0%) in Steady-High, 8 (4.4%) in Disrupted-Rhythmicity, and 2 (1.4%) in Partial-Recovery (*p = 0.04*).

#### One-Year Outcomes

Functional independence persisted in 83 (56.0%) Partial-Recovery, 71 (39.5%) Disrupted-Rhythmicity, and 70 (34.7%) Steady-High (*p* < 0.001). In multivariable analysis adjusted for the expanded covariate set, Steady-High (aOR 0.44; 95% CI 0.25–0.79; *p* = 0.005) and Disrupted-Rhythmicity (aOR 0.53; 95% CI 0.29–0.95; *p* = 0.03) remained independently associated with reduced odds of favorable outcome. One-year mortality was highest in Steady-High (37 [18.4%]), intermediate in Disrupted-Rhythmicity (22 [12.3%]), and lowest in Partial-Recovery (9 [6.0%]; *p* = 0.01).

#### In Cox Proportional-Hazards Analyses

mortality risk differed significantly across BP phenotypes. Compared with Partial-Recovery, hazard ratios for all-cause mortality were 3.28 (95% CI 1.52–7.06; *p* = 0.003) for Steady-High and 2.14 (95% CI 1.01–4.55; *p* = 0.047) for Disrupted-Rhythmicity. These associations remained directionally consistent after multivariable adjustment for age, sex, baseline NIHSS, hypertension, diabetes, atrial fibrillation, and reperfusion treatment (Table S10).

#### Cardiovascular Outcomes

**Fig. 4:**
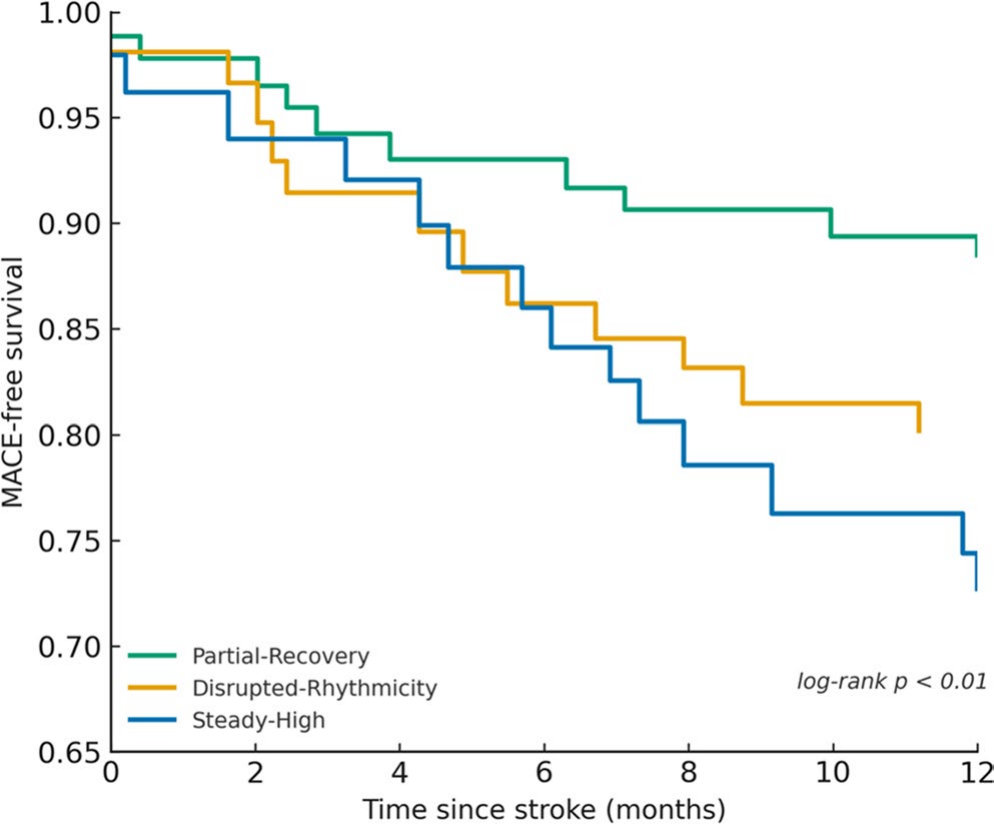
Kaplan–Meier estimates of MACE-free survival over 12 months according to circadian blood pressure phenotypes. Patients in the Partial-Recovery group had the highest MACE-free survival, Disrupted-Rhythmicity intermediate, and Steady-High the lowest (log-rank *p* < 0.01)

Major adverse cardiovascular events (MACE) occurred in 55 (27.3%) Steady-High, 36 (19.8%) Disrupted-Rhythmicity, and 17 (11.5%) Partial-Recovery (*p = 0.004*). Kaplan–Meier analysis confirmed these differences (12-month MACE-free survival = 72.7%, 80.2%, and 88.5%; log-rank *p* < 0.01; Fig. 4).

**Fig. 5:**
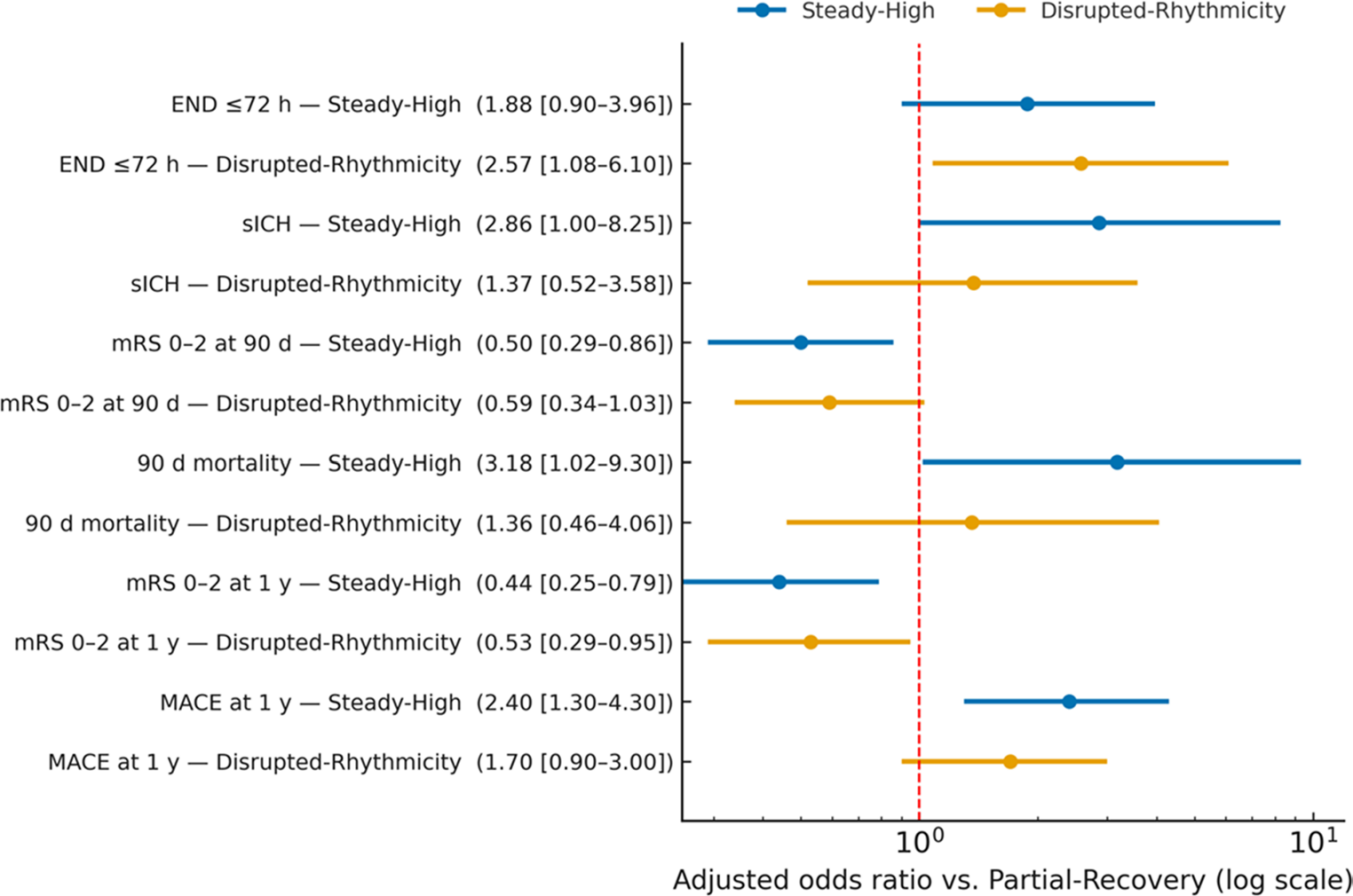
Associations of circadian blood-pressure phenotypes with early and long-term outcomes. Forest plot of adjusted associations by phenotype (reference: Partial-Recovery) for END ≤ 72 h, sICH after reperfusion, 90-day mRS 0–2, 90-day mortality, 1-year mRS 0–2, and 1-year MACE

In multivariable analysis, Steady-High was associated with a significantly higher risk of MACE (aOR 2.4, 95% CI 1.3–4.3, *p* = 0.005), whereas Disrupted-Rhythmicity showed a non-significant intermediate increase (aOR 1.7, 95% CI 0.9–3.0, *p* = 0.08). The difference between the two phenotypes was not statistically significant (p for comparison = 0.29). A forest plot summarising adjusted associations with acute and long-term outcomes is shown in Fig. 5.

### Risk Factor Profiles of Early BP Phenotypes

Baseline characteristics differed significantly across the three circadian blood-pressure phenotypes (Table 4). Patients in the Steady-High cluster were older (median 76 years; IQR 69–82; *p* < 0.001) and had the highest burden of vascular risk factors, including hypertension (163 [81.2%] vs. 128 [71.0%] and 102 [68.9%]; *p* < 0.001), obesity (105 [52.4%] vs. 73 [40.3%] and 57 [38.5%]; *p* = 0.01), diabetes (66 [32.7%] vs. 47 [26.1%] and 33 [22.3%]; *p* = 0.04), and coronary artery disease (64 [31.8%] vs. 42 [23.4%] and 32 [21.6%]; *p* = 0.03). The Disrupted-Rhythmicity cluster comprised relatively younger patients (median 69 years; IQR 62–77) with fewer traditional risk factors but a higher proportion of cryptogenic/ESUS strokes (74 [41.2%] vs. 53 [28.3%] and 28 [18.9%]; *p* < 0.01). The Partial-Recovery phenotype showed the most favorable vascular profile and the highest proportion of lacunar infarcts (66 [44.6%]; *p* = 0.02). In multivariable analysis, hypertension independently predicted Steady-High membership (OR 2.4; 95% CI 1.5–3.9; *p* < 0.001), and obesity was associated with Steady-High and Disrupted-Rhythmicity (OR 1.6; 95% CI 1.1–2.5; *p* = 0.02).

**Table 4:** Baseline risk factor profiles across early BP phenotypes

Characteristic	Steady-High. (*n* = 201, 38%)	Disrupted-Rhythmicity (*n* = 180, 34%)	Partial-Recovery (*n* = 148, 28%)	*p*-value
Demographics				
Age, median (IQR), y	76 (69–82)	69 (63–75)	68 (61–74)	< 0.001
Female sex	93 (46.3)	72 (40.0)	65 (43.9)	0.21
Vascular risk factors				
Hypertension	163 (81.1)	128 (71.1)	102 (68.9)	< 0.001
Diabetes mellitus	66 (32.8)	47 (26.1)	33 (22.3)	0.04
Obesity	105 (52.2)	73 (40.6)	57 (38.5)	0.01
Hypercholesterolemia	77 (38.3)	68 (37.8)	58 (39.2)	0.91
Smoking	50 (24.9)	49 (27.2)	40 (27.0)	0.77
Cardiovascular disease				
Coronary artery disease	64 (31.8)	42 (23.3)	32 (21.6)	0.03
Heart failure	24 (11.9)	15 (8.3)	10 (6.8)	0.14
Atrial fibrillation	54 (26.9)	38 (21.1)	29 (19.6)	0.08

Values are* n (%) *unless otherwise indicated. Continuous variables are presented as median (IQR). Group comparisons were performed using χ² or Kruskal–Wallis tests, as appropriate; for categorical variables with expected cell counts < 5, Fisher’s exact test was applied

### Effect Modifiers of Phenotype–Outcome Associations

We next examined whether the associations between circadian blood-pressure phenotypes and clinical outcomes were modified by treatment modality, sex, or adiposity. Among patients who underwent EVT, the Steady-High phenotype was associated with a higher risk of symptomatic intracerebral hemorrhage compared with those without EVT (aOR, 3.6; 95% CI, 1.3–10.3; *p* = 0.01; *p* for interaction = 0.04). This effect modification was not observed for IVT, where the associations between BP phenotypes and outcomes remained consistent with the overall cohort. Sex-stratified analyses showed that female patients with the Steady-High phenotype had a greater likelihood of poor 90-day outcome (aOR, 2.0; 95% CI, 1.0–4.1; *p* = 0.04; *p* for interaction = 0.05), independent of age and baseline NIHSS. Adiposity further modified these associations: obese patients in the Steady-High cluster had the lowest probability of achieving functional independence (aOR, 2.3; 95% CI, 1.0–5.2; *p* = 0.04; *p* for interaction = 0.05), whereas the Partial-Recovery phenotype conferred the greatest relative benefit in non-obese patients (Table 5).

**Table 5:** Effect modification of circadian BP phenotypes by treatment, sex, and adiposity

Effect modifier	Phenotype (ref = Partial-Recovery)	Adjusted OR (95% CI)	*p*-value	*p* for interaction
Treatment				
EVT vs. none	Steady-High → sICH	3.6 (1.3–10.3)	0.01	0.04
	Disrupted-Rhythmicity → END ≤ 72 h	2.5 (1.0–5.8)	0.04	0.13
IVT vs. none	Steady-High → sICH	2.6 (0.8–7.9)	0.09	0.23
Sex				
Female (vs. male)	Steady-High → poor mRS90	2.0 (1.0–4.1)	0.04	0.05
	Disrupted-Rhythmicity → END ≤ 72 h	1.3 (0.6–2.8)	0.31	0.19
Adiposity				
Obese (vs. non-obese)	Steady-High → poor mRS90	2.3 (1.0–5.2)	0.04	0.05
	Partial-Recovery → favorable mRS90	0.8 (0.4–1.4)	0.27	0.06

Models adjusted for age, sex (except in sex-stratified analyses), baseline NIHSS, hypertension, glycemic status (diabetes, admission glucose, and HbA1c), atrial fibrillation, and treatment modality (IVT and/or EVT as applicable). Interaction terms tested the phenotype × modifier effects within multivariable logistic regression models. Reference category: Partial-Recovery phenotype. Abbreviations: *END *early neurological deterioration (≥ 4-point NIHSS increase within 72 h or death),* sICH *symptomatic intracerebral hemorrhage (Heidelberg definition),* EVT *endovascular treatment,* IVT *intravenous thrombolysis

### Renal Dysfunction as a Modifier of Early Circadian Blood Pressure Dynamics

To account for systemic comorbidities potentially affecting blood-pressure regulation, a prespecified subgroup analysis was performed by chronic kidney disease (CKD) status, defined as an estimated glomerular filtration rate < 60 mL/min/1.73 m² or documented renal impairment before stroke. Among 529 patients, 148 (28.0%) had CKD. These patients were older (mean, 72.4 ± 9.1 years vs. 66.8 ± 10.3 years; *p* < 0.001), more often female (88 [59.5%] vs. 147 [38.6%]; *p* = 0.002), and more frequently diabetic (92 [62.2%] vs. 125 [32.8%]; *p* < 0.001). Across 72 h, CKD was associated with higher mean systolic BP (148.3 ± 17.5 mm Hg vs. 141.5 ± 15.9 mm Hg; *p* = 0.03) and a smaller nocturnal decline (− 6.1 ± 4.8 mm Hg vs. −9.8 ± 5.1 mm Hg; *p* = 0.02). Non-dipping and reverse-dipping patterns were more frequent in CKD (105 [70.9%] vs. 224 [58.8%] on night 3; *p* = 0.05). The Steady-High phenotype predominated (70 [47.3%] vs. 131 [34.4%]; *p* = 0.02), whereas Partial-Recovery was less common (22 [14.9%] vs. 126 [33.1%]; *p* < 0.001). Clinical outcomes were poorer in CKD, with higher rates of early neurological deterioration (27 [18.2%] vs. 39 [10.2%]; *p* = 0.04), 90-day mortality (29 [19.6%] vs. 32 [8.4%]; *p* = 0.01), and 1-year MACE (41 [27.7%] vs. 49 [12.9%]; *p* = 0.002). In multivariable mixed-effects models adjusted for age, sex, baseline NIHSS, hypertension, diabetes, atrial fibrillation, and reperfusion therapy, CKD remained associated with higher mean systolic BP (β = +5.4 mm Hg; 95% CI, 1.1–9.6; *p* = 0.01) and reduced circadian amplitude (β = −2.8 mm Hg; 95% CI, − 5.1 to − 0.6; *p* = 0.014). Adjustment for CKD did not alter the associations between BP phenotypes and outcomes, and no significant interactions were detected (Table 6).

**Table 6:** Comparison of patients with and without chronic kidney disease (CKD)

Parameter	Non-CKD (*n* = 381)	CKD (*n* = 148)	*p* value
Age, y, mean ± SD	66.8 ± 10.3	72.4 ± 9.1	< 0.001
Female sex	147 (38.6)	88 (59.5)	0.002
Diabetes mellitus	125 (32.8)	92 (62.2)	< 0.001
Baseline NIHSS, median [IQR]	8 [4–13]	10 [6–16]	0.06
Mean SBP (0–72 h), mmHg	141.5 ± 15.9	148.3 ± 17.5	0.03
Nocturnal BP decline, mmHg	−9.8 ± 5.1	−6.1 ± 4.8	0.02
Dipping pattern night 1 (D/N/R)	122 (32.0)/179 (47.0)/80 (21.0)	32 (21.6)/81 (54.7)/35 (23.7)	0.08
Dipping pattern night 3 (D/N/R)	157 (41.2)/160 (42.0)/64 (16.8)	43 (29.1)/74 (50.0)/31 (20.9)	0.05
Steady-High phenotype	131 (34.4)	70 (47.3)	0.02
Disrupted-Rhythmicity phenotype	124 (32.5)	56 (37.8)	0.28
Partial-Recovery phenotype	126 (33.1)	22 (14.9)	< 0.001
Early neurological deterioration ≤ 72 h	39 (10.2)	27 (18.2)	0.04
Symptomatic ICH	23 (6.0)	13 (8.8)	0.31
Favourable outcome (mRS 0–2 90 d)	208 (54.6)	61 (41.2)	0.009
Mortality (in-hospital/90 d)	32 (8.4)	29 (19.6)	0.01
MACE (1-year)	49 (12.9)	41 (27.7)	0.002

Values are n (%) unless stated otherwise; continuous data are mean ± SD or median [IQR]. D = dipper (≥ 10 % nocturnal decline); N = non-dipper (< 10 % decline); R = reverse-dipper (nocturnal rise). Abbreviations: *d* day; *y* year; *ICH* intracerebral hemorrhage; *BP* blood pressure, *SBP* systolic blood pressure; *MACE* major adverse cardiovascular events (death, recurrent stroke, or myocardial infarction within 1 year)

### Stroke Subtype Analysis

Exploratory analyses stratified by stroke subtype (ESUS *n* = 98; cardioembolic *n* = 209; large-artery atherosclerosis *n* = 110; lacunar *n* = 40) revealed distinct patterns of circadian blood-pressure disorganization (see Table S9 for extended multivariable models). In ESUS, the Disrupted-Rhythmicity phenotype was associated with higher odds of early neurological deterioration (aOR 2.8, 95% CI 1.2–6.4; *p* = 0.01). In cardioembolic stroke, circadian disorganization was linked to an increased risk of symptomatic intracerebral hemorrhage after reperfusion (aOR 3.1, 95% CI 1.1–8.7; *p* = 0.03). Among patients with large-artery atherosclerosis, the Steady-High phenotype predicted poor 90-day functional outcome (aOR 2.2, 95% CI 1.0–4.6; *p* = 0.04). Lacunar stroke cases displayed relatively preserved circadian organization, with the highest proportion of physiological dippers and the lowest prevalence of adverse BP phenotypes. In extended multivariable models including infarct volume (per 10-mL increase), baseline NIHSS, and glycemic status, the phenotype–outcome associations remained stable. Additional adjustment for vascular territory, onset-to-treatment times, and reperfusion therapy (intravenous thrombolysis 15% and endovascular treatment 8%) did not materially alter effect estimates for early neurological deterioration (ΔaOR < 0.10), symptomatic intracerebral hemorrhage (ΔaOR < 0.08), or favorable 90-day outcome (ΔaOR < 0.09; all *p* > 0.10). No significant phenotype × treatment interactions were observed after false-discovery-rate correction, indicating robust associations across stroke subtypes.

### Sensitivity and Robustness Analyses

Findings were consistent across all prespecified sensitivity analyses (Tables S1–S6; Figures S1–S3). Results remained unchanged when restricted to patients with high-density monitoring, after multiple imputation, or when cosinor parameters were replaced by conventional variability indices. Alternative night definitions, exclusion of arterial-line data, adjustment for acute-care exposures, and winsorizing of outliers yielded comparable estimates. Model refinements—including additional covariates, propensity weighting, and correction for multiple testing—produced no significant deviation from the main results. Cluster stability was high: a three-cluster solution consistently provided optimal fit, and alternative cluster numbers did not improve model performance. Exclusion of TIA or hemorrhagic transformations, and restriction to imaging-confirmed infarction, yielded similar estimates. Inclusion of chronic kidney disease as a covariate or subgroup factor shifted circadian profiles as expected but left the direction and significance of phenotype–outcome associations unchanged.

### Relationship Between BP Trajectories and Infarct Volume

Infarct volume data were available in 359 of 529 patients (68%). Median infarct volume differed significantly across BP phenotypes (*p* = 0.01): non-dipping or reverse-dipping profiles were associated with larger infarct volumes (52 mL [IQR 31–78]) compared with Partial-Recovery profiles (34 mL [IQR 20–55]; Δ = 18 mL, 95% CI 5–30). Among patients undergoing EVT (*n* = 42; 8%), > 20% periprocedural reductions in systolic BP were linked to greater infarct volume (+ 18 mL, 95% CI 7–28; *p* = 0.002). Adjustment for infarct volume in multivariable models did not materially change the direction or magnitude of phenotype–outcome associations (aOR 1.42, 95% CI 1.10–1.83; *p* = 0.007).

### Additional Adjustment for Imaging and Treatment Covariates

To assess robustness, extended multivariable logistic-regression models sequentially incorporated infarct volume, vascular territory, onset-to-treatment interval, reperfusion parameters (IVT, EVT, final eTICI), and collateral grade (EVT subset). Associations between circadian BP phenotypes and clinical outcomes remained stable (all ΔaOR < 10%; all *p* ≥ 0.10). Infarct volume, available in 68% of patients, correlated strongly with baseline NIHSS (*r* = 0.71; *p* < 0.001) but was not independently predictive when both variables were included. Substituting infarct volume for NIHSS yielded nearly identical estimates (ΔaOR < 8%). Inclusion of vascular territory, etiologic subtype, or treatment variables (IVT 15%, EVT 8%) produced comparable results. Multicollinearity diagnostics showed VIF < 2.0 for all covariates, and no significant phenotype × treatment or phenotype × sex interactions were detected after false-discovery-rate correction. Detailed coefficients are provided in Supplementary Table S9.

### Comparison with Hypertensive Non-Stroke Populations

In hypertensive cohorts from the MAPEC study [50] and ABC-H meta-analysis [51, 52], the mean nocturnal systolic BP decline was 10–12%, with non-dipping and reverse-dipping present in 33.6% of participants (pooled estimate). In our acute stroke cohort, the mean nocturnal systolic decline was 4.3 ± 7.8%, and 72.4% (95% CI 68.1–76.4) of patients exhibited either non-dipping or reverse-dipping trajectories. The combined prevalence of abnormal dipping was significantly higher than in the pooled hypertensive reference data (Z = 16.8, *p* < 0.001; Cohen’s h = 0.91, indicating a large standardized difference). The mean nocturnal decline was likewise reduced compared with hypertensive populations (t = − 14.2, *p* < 0.001). Across stroke subtypes, non-dipping and reverse-dipping remained more frequent than in hypertensive references (*p* < 0.001 for all χ² tests). After adjustment for age, sex, pre-existing hypertension, diabetes, and baseline systolic pressure, stroke onset remained independently associated with reduced nocturnal amplitude (β = −0.42, 95% CI − 0.53 to − 0.31, *p* < 0.001).

## Discussion

In this large, prospective cohort with high-density blood pressure monitoring, we provide the first evidence that circadian blood pressure organization after ischemic stroke follows distinct, subtype-specific trajectories with major prognostic implications. Earlier descriptive studies reported loss of nocturnal dipping and disruption of diurnal blood pressure rhythmicity after stroke [53–56]. Our work builds on these foundational studies by applying high-density, multiscale analyses to delineate reproducible, subtype-specific circadian phenotypes within the first 72 h after ischemic stroke. Within this period, patients exhibited profound disruption of amplitude, delayed acrophase, and a predominance of non-dipping and reverse-dipping patterns. These abnormalities were not uniform but varied systematically across etiologies: ESUS showed the most pronounced amplitude loss and highest prevalence of reverse dipping, cardioembolic strokes were dominated by non-dipping, large-artery atherosclerosis was characterized by persistently elevated nocturnal pressure, and lacunar strokes maintained comparatively preserved rhythmicity. By applying unsupervised clustering to multiscale variability features, we uncovered three reproducible phenotypes—*Steady-High*,* Disrupted-Rhythmicity*,* and Partial-Recovery*—that independently predicted early deterioration, hemorrhagic complications, mortality, and functional recovery. These findings establish circadian blood pressure organization as a dynamic biomarker domain in acute stroke and open new opportunities for individualized risk stratification. Our results extend and refine prior evidence on the relationship between blood pressure and stroke subtypes. Epidemiological and genetic studies have consistently shown that hypertension is most strongly associated with deep intracerebral hemorrhage and lacunar infarcts, less so with large-artery atherosclerosis, and least with cardioembolic stroke [14, 57, 58]. Clinical observations further suggested that patients with intracerebral hemorrhage or lacunar infarcts present with the highest systolic blood pressure, while cardioembolic strokes often show lower admission levels [59–62]. Our analysis goes beyond these static descriptions by demonstrating that ischemic stroke subtypes differ not only in baseline pressure but in the circadian dynamics of the first three nights, with direct consequences for outcome. The observation that ESUS patients experienced the most profound loss of rhythmicity aligns with reports linking cryptogenic stroke to autonomic imbalance and concealed embolic risk [63]. Likewise, the predominance of non-dipping in cardioembolic stroke resonates with evidence of reduced autonomic resilience in atrial fibrillation [64, 65]. In our cohort, the majority of strokes occurred between 5:00 and 10:00 AM, corresponding to the circadian window of heightened cerebrovascular vulnerability described in prior population studies [66, 67]. This clustering within the morning surge phase likely reflects the interaction between intrinsic rhythmicity of vascular tone, sympathetic activation, and the steep rise in arterial pressure after awakening. Importantly, these dynamics may not only precipitate stroke onset but also modulate early post-stroke blood pressure trajectories: patients presenting during the morning surge exhibited more frequent non-dipping or reverse-dipping profiles within the first 72 h, suggesting that the endogenous circadian phase at symptom onset can shape the subsequent hemodynamic organization.

A key innovation of our study lies in moving beyond conventional variability metrics such as standard deviation or coefficient of variation [10]. By leveraging cosinor regression, entropy measures, and unsupervised clustering, we identified clinically meaningful phenotypes that captured cerebrovascular vulnerability more effectively than traditional indices. The Steady-High phenotype, marked by persistently elevated nocturnal pressure, was strongly associated with hemorrhagic complications after reperfusion and excess mortality. This is biologically plausible: sustained hypertension and absent dipping may aggravate blood–brain barrier fragility and microvascular stress, thereby amplifying the risk of symptomatic intracerebral hemorrhage [68, 69]. The Disrupted-Rhythmicity phenotype, characterized by blunted amplitude and reverse dipping, predicted early neurological deterioration, particularly in ESUS, suggesting that dynamic instability rather than absolute levels can precipitate recurrent embolization, impaired collateral flow, or metabolic compromise in ischemic tissue. This interpretation aligns with prior observations that both periprocedural hypotension and post-recanalization BP variability are associated with infarct expansion and poorer functional recovery after large-vessel occlusion stroke [40, 42]. In our cohort, patients with non-dipping or reverse-dipping trajectories exhibited larger infarct volumes, supporting the concept that early circadian BP disorganization may aggravate ischemic injury through impaired perfusion stability and loss of autoregulatory control. The Partial-Recovery phenotype, enriched in lacunar stroke, showed progressive restitution of rhythmicity and conferred the highest likelihood of functional independence, consistent with microangiopathic substrates being less prone to circadian disintegration. The mechanistic framework emerging from our results integrates systemic autonomic dysfunction, vascular substrate, and metabolic modulation. Acute stroke perturbs hypothalamic centers, neurohumoral circuits, and baroreflex function, leading to sympathetic overdrive and impaired circadian regulation [68, 70, 71]. Subtype-specific mechanisms seem to shape these trajectories [72]. In large-artery atherosclerosis, chronic arterial stiffness and endothelial dysfunction may underlie the Steady-High profile [73, 74]. In ESUS, unstable embolic sources and impaired neurocardiac integration may predispose to Disrupted-Rhythmicity, whereas preserved autoregulation may protect patients with lacunar stroke.

Major circadian regulators—cortisol, melatonin, and coagulation rhythms—further modulate blood pressure trajectories and stroke outcomes. These systems are coordinated by the central clock in the suprachiasmatic nucleus and peripheral clock genes, synchronizing neurohumoral and vascular function across the 24-hour cycle [75, 76]. Cortisol peaks in the early morning, and exaggerated post-stroke elevations have been linked to heightened neuroinflammation, impaired recovery, and worse neurological outcomes, particularly in older patients [77, 78]. Conversely, blunted nocturnal melatonin secretion diminishes antioxidative and anti-inflammatory protection, promoting circadian desynchrony and sleep disruption [79, 80]. Morning surges in prothrombotic mediators such as plasminogen activator inhibitor 1 (PAI-1) and fibrinogen may exacerbate vascular vulnerability and reperfusion injury [81, 82]. Dysregulation of these hormonal and hemostatic oscillations likely amplifies BP instability and contributes to the adverse outcomes observed in non-dipping and reverse-dipping phenotypes.

Beyond endocrine clocks, several systemic pathways plausibly drive the circadian BP disorganization observed in our cohort [83]. The autonomic nervous system (ANS), under suprachiasmatic control, orchestrates day–night fluctuations in heart rate and vascular tone; stroke-related disruption of hypothalamic and insular circuitry, together with baroreflex dysfunction, attenuates nocturnal dipping and amplifies short-term BP lability [77, 84, 85]. These alterations align with the predominance of non-dipping and reverse-dipping patterns we identified in ESUS and cardioembolic stroke.

Obstructive sleep apnea (OSA), characterized by intermittent hypoxia and arousal-related sympathetic surges, further destabilizes autonomic and vascular rhythms and shifts the expression of core clock genes (BMAL1, CLOCK, PER1, CRY1), reinforcing blunted or inverted BP profiles [86–90]. Such sleep-related desaturations were documented in our cohort and may partially account for the pronounced nocturnal dysregulation observed in the Steady-High and Disrupted-Rhythmicity phenotypes. In parallel, inflammatory–clock crosstalk (NF-κB–mediated suppression of BMAL1/PER2) blunts diurnal vascular reactivity and contributes to endothelial dysfunction (Curtis et al., 2014; Shen et al., 2021), while endothelial nitric oxide synthase (eNOS) normally exhibits a circadian rhythm promoting daytime vasodilation and nocturnal pressure decline [91–94]. Loss of this rhythm through oxidative stress and eNOS uncoupling may further perpetuate the sustained nocturnal hypertension typical of the Steady-High phenotype. This vascular rigidity and redox imbalance likely interact with systemic metabolic cues, linking endothelial stress to broader autonomic and hormonal dysregulation.

Building on our previous findings that adiposity and metabolic dysregulation can promote covert atrial fibrillation and thus cardioembolic risk after stroke [6, 95], the present study extends this framework to circadian BP regulation. Obesity similarly modified vascular rhythmicity [96], aggravating the adverse impact of Steady-High BP organization on outcome. This convergence of autonomic, metabolic, and endothelial pathways suggests a shared substrate linking atrial excitability, vascular stiffness, and loss of temporal vascular coherence after stroke. Such multidimensional dysregulation may not only underlie acute hemodynamic instability but also imprint long-term trajectories of cerebrovascular recovery and resilience.

A key strength of our study lies in the demonstration that circadian BP phenotypes not only predict early deterioration and 90-day outcomes but also remain strongly associated with functional recovery, mortality, and recurrent vascular events at one year. Prior studies of BP variability in acute stroke were largely limited to short-term horizons, did not establish durable prognostic value or performed a subtype specific analysis [10, 69]. Our findings therefore position circadian BP organization as a long-term biomarker of cerebrovascular vulnerability rather than a transient epiphenomenon.

The translational implications of these findings are substantial. First, circadian blood pressure monitoring provides a readily available, non-invasive biomarker for early risk stratification [97, 98]. Identification of patients with Steady-High or Disrupted-Rhythmicity patterns could inform intensified monitoring, personalized blood pressure management, and more cautious reperfusion strategies. Second, therapeutic interventions may need to be tailored not only by stroke subtype but also by circadian phenotype: for instance, aggressive blood pressure lowering may be hazardous in ESUS or cardioembolic patients with disrupted rhythmicity, while targeting nocturnal hypertension may diminish hemorrhagic complications in large-artery atherosclerosis. Third, sex and obesity emerged as modifiers of risk, underscoring the necessity of individualized treatment approaches and the design of future trials that explicitly account for these subgroups. Finally, our findings highlight circadian biology as a therapeutic target [77]. Restoration of nocturnal dipping patterns has shown cardiovascular benefit [13, 99], and testing such strategies in acute stroke may represent a novel intervention paradigm. The predominance of morning stroke onset in our cohort aligns with the well-known circadian peak of cerebrovascular vulnerability. Adjustment for onset phase did not attenuate the associations between circadian BP phenotypes and outcome, indicating that the observed patterns are not merely a reflection of stroke timing. Likewise, the fixed ward schedule of antihypertensive administration (06:00, 12:00, 18:00 h) and occasional nighttime doses did not influence phenotype–outcome relationships.

Compared with hypertensive reference populations, circadian disruption after stroke was substantially greater (72% vs. 33%, *p* < 0.001), with a markedly reduced nocturnal decline (4% vs. 11%) [100]. These differences exceed the range of chronic hypertensive variability and likely reflect an acute neurogenic mechanism rather than baseline vascular stiffness. Several avenues for future research arise. Longitudinal analyses should clarify whether early circadian disruption persists into subacute and chronic phases and whether its restitution mediates recovery. Integration of ambulatory monitoring, wearable sensors, and biomarkers of autonomic function could refine phenotyping and enhance mechanistic insight. Interventional trials should test whether circadian phenotype-guided blood pressure targets improve outcomes, particularly in high-risk groups such as ESUS and cardioembolic stroke. Finally, linking circadian phenotypes with neuroimaging markers of collateral flow, microvascular integrity, and blood–brain barrier permeability may identify structural correlates of these dynamic patterns. In conclusion, this study demonstrates that circadian blood pressure organization is not merely disrupted after ischemic stroke but follows reproducible, subtype-specific trajectories that carry major prognostic significance. By applying advanced analytic methods in a large, prospective cohort, we introduce circadian blood pressure phenotyping as a novel, clinically actionable framework for individualized stroke care and as a potential target for future therapeutic intervention. Given the exploratory nature of this observational study, our analyses indicate associations rather than direct causality. Nonetheless, the consistent and biologically plausible patterns observed suggest a meaningful pathophysiological link that merits confirmation in mechanistic and interventional research.

## Limitations

This single-center observational design limits causal inference and generalizability. Blood pressure acquisition was heterogeneous, with arterial lines and automated cuffs at variable intervals, and interpolation of gaps may have biased variability estimates despite sensitivity analyses. While our analyses demonstrate consistent associations between circadian blood pressure organization and clinical outcomes, they do not establish causality. The exploratory nature of the study precludes determination of whether circadian disruption contributes to adverse outcomes or reflects the severity of neurological injury. Circadian modelling assumed a fixed 24-hour rhythm and relied on ward night periods rather than individual biological timing, which may have led to misclassification of amplitude, acrophase, and dipping status. Treatment exposures, including reperfusion therapies, sedatives, vasopressors, and antihypertensives, were not randomized, leaving potential for residual confounding. Stroke subtype classification, particularly embolic stroke of undetermined source, may be imperfect without uniform prolonged monitoring and extended cardiac or aortic evaluation. Subgroup sizes, especially for lacunar stroke and certain phenotype–subtype combinations, were modest, limiting statistical power for interaction analyses. Additionally, time series were aligned to admission rather than symptom onset or circadian phase, and pre-stroke dipping status and autonomic function were not assessed. Finally, Sleep apnea was not systematically assessed; although nocturnal desaturations were recorded, they were not included in the analyses due to potential confounding by environmental, respiratory, metabolic, and neurogenic factors in the acute stroke setting, which may themselves influence BP trajectories. The potential influence of infarct location on circadian blood pressure organization—particularly lesions involving autonomic control regions such as the insula, anterior cingulate cortex, hypothalamus, and brainstem nuclei (nucleus tractus solitarius, rostral ventrolateral medulla)—was beyond the scope of the present analysis and warrants investigation in future studies integrating standardized lesion mapping and autonomic biomarkers.

## Conclusions

Circadian blood pressure rhythms are profoundly disrupted during the first 72 h after acute ischemic stroke and follow distinct, subtype-specific patterns. Exploratory, data-driven clustering identified three early phenotypes with consistent prognostic associations, spanning adverse acute and long-term outcomes to trajectories of recovery and independence. These findings highlight circadian blood pressure organization as a promising translational marker linking vascular chronobiology to individualized hemodynamic management in acute stroke. Integrating circadian profiling into early monitoring frameworks may advance personalized care and guide future chronotherapeutic strategies.

## Supplementary Information

Below is the link to the electronic supplementary material.

Supplementary File 1 (DOCX 878 KB)

## Data Availability

The datasets generated and/or analyzed during the current study are not publicly available due to institutional data protection regulations but are available from the corresponding author upon reasonable request and after approval by the institutional data protection officer.
