# Supplementary material for: Circadian Blood Pressure Phenotyping Identifies Subtype-Specific Risk and Outcomes in Acute Ischemic Stroke: A Prospective Study
Source: Transl Stroke Res. 2025 Dec 8;17(1):5. doi: 10.1007/s12975-025-01400-x (PMC12682914; doi:10.1007/s12975-025-01400-x)

**Supplement**

**Table S1. High measurement density and missing data** Analyses restricted to patients with high blood-pressure measurement density and analyses after multiple imputation of missing data.

| Analysis | N (subset) | Findings vs. main analysis | Effect on END / sICH / 90-day mRS |
| --- | --- | --- | --- |
| High BP measurement density (≥80% of expected readings, nights 1–3) | 412 (78%) | Same 3 phenotypes; phenotype membership variation <8% | Point estimates Δ <10%; all previously significant associations remained significant |
| Multiple imputation (m=20) for missing BP segments and covariates | 529 (100%) | Estimates concordant with complete-case analysis | No material change; phenotype–outcome associations preserved |

**Abbreviations:** BP, blood pressure; END, early neurological deterioration; sICH, symptomatic intracerebral hemorrhage; mRS, modified Rankin Scale.

**Table S2. Sensitivity analyses with alternative BP variability metrics**

| Metric | END ≤72 h (aOR, 95% CI) | sICH (aOR, 95% CI) | 90-d mRS 0–2 (aOR, 95% CI) | Effect vs. cosinor |
| --- | --- | --- | --- | --- |
| SD | 2.6 (1.1–6.1) | 2.9 (1.0–8.4) | 0.52 (0.30–0.91) | Associations reproduced |
| CV | 2.7 (1.2–6.2) | 3.0 (1.1–8.6) | 0.50 (0.29–0.88) | Associations reproduced |
| ARV | 2.5 (1.0–5.9) | 2.8 (0.9–8.2) | 0.55 (0.31–0.95) | Associations reproduced |
| MESOR+Amplitude+ARV | 2.4 (1.0–5.7) | 2.7 (0.9–8.1) | 0.57 (0.32–1.00) | Minimal attenuation |

**Abbreviations:** MESOR, midline estimating statistic of rhythm; ARV, average real variability; aOR, adjusted odds ratio; CI, confidence interval.

**Table S3. Night definition and measurement modality**

| Analysis | Phenotype membership variation | Effect on outcomes |
| --- | --- | --- |
| Night 22:00–06:00 (primary) | — | Reference |
| Night 23:00–07:00 | <8% | aORs for END/sICH Δ <10% |
| Actigraphy-based rest periods | <8% | aORs for END/sICH Δ <10% |
| Restrict to invasive monitoring only | <3% | Effect sizes stable |
| Exclude invasive monitoring | <3% | Effect sizes stable |

**Table S4. Outliers and acute-care exposure**

| Analysis | Findings | Effect on outcomes |
| --- | --- | --- |
| Winsorize SBP at 1st/99th percentiles | Maximal Δ <5% in effect sizes | Stable |
| Exclude <12 h monitored time per night | N reduced by 8% | Conclusions unchanged |
| Adjust for sedation, fever, early complications | Coefficients shifted <10% | No material change |

**Table S5. Model specification and interaction terms**

| Analysis | Findings | Effect on outcomes |
| --- | --- | --- |
| Add/pre-specify covariates (age, sex, NIHSS, HTN, DM, AF, reperfusion) | Coefficient shifts <10% | No material change |
| Propensity-score weighting for EVT/IVT | aORs within 95% CI of main estimates | Results robust |
| FDR correction for multiple outcome tests | All key associations retained | Results robust |
| Interaction phenotype×EVT | Effect direction retained; nominal significance unchanged | Consistent with main text |
| Interaction phenotype×sex | Effect direction retained; nominal significance unchanged | Consistent with main text |
| Interaction phenotype×obesity | Effect direction retained; nominal significance unchanged | Consistent with main text |

**Abbreviations:** NIHSS, National Institutes of Health Stroke Scale; HTN, hypertension; DM, diabetes mellitus; AF, atrial fibrillation; EVT, endovascular therapy; IVT, intravenous thrombolysis; FDR, false discovery rate; aOR, adjusted odds ratio; CI, confidence interval.

**Table S6. Clustering stability and population sensitivity**

| Analysis | Findings | Effect on outcomes |
| --- | --- | --- |
| Model selection by BIC | 3-cluster solution consistently favored | Robust; aORs unchanged |
| Bootstrapped re-clustering | Adjusted Rand index >0.80 | Stable phenotype allocation |
| 10-fold leave-subset-out procedure | Adjusted Rand index >0.80 | Stable phenotype allocation |
| Force 2-cluster solution | Steady-High + Disrupted merged; BIC not improved | Associations attenuated |
| Force 4-cluster solution | Partial-Recovery split; BIC not improved | No strengthening of associations |
| Exclude TIAs | N reduced by 5%; estimates within 95% CI of main | Stable |
| Exclude hemorrhagic transformations | N reduced by 7%; estimates within 95% CI of main | Stable |
| Restrict to ischemic strokes with imaging-confirmed infarction | N reduced by 9%; effect sizes Δ <10% | Stable |
| Subtype-specific signals (ESUS × reverse-dipping; cardioembolic × BP Instability) | Direction and magnitude preserved | Robust |

**Abbreviations:** BIC, Bayesian information criterion; ARI, adjusted Rand index; ESUS, embolic stroke of undetermined source; TIA, transient ischemic attack; aOR, adjusted odds ratio; CI, confidence interval.

**Table S7. Antihypertensive therapy at baseline by circadian BP phenotype** Table displays proportion of patients receiving any antihypertensive medication, number of agents, and administration window (scheduled 06:00/12:00/18:00 vs. any nighttime 22:00–05:59) as well as cumulative nighttime exposure during nights 1–3. Percentages are shown for the total cohort and by circadian BP phenotype.

| Antihypertensive therapy | Steady-High (n=271) | Disrupted-Rhythmicity (n=243) | Partial-Recovery (n=200) | p-value |
| --- | --- | --- | --- | --- |
| No antihypertensive therapy | 41 (15.1%) | 61 (25.1%) | 60 (30.0%) | <0.001 |
| Monotherapy | 76 (28.0%) | 64 (26.3%) | 58 (29.0%) | 0.72 |
| Combination (≥2 agents) | 154 (56.8%) | 118 (48.6%) | 82 (41.0%) | <0.001 |
| ACE inhibitors / ARBs | 138 (50.9%) | 111 (45.7%) | 78 (39.0%) | 0.02 |
| Beta-blockers | 121 (44.6%) | 95 (39.1%) | 66 (33.0%) | 0.03 |
| Calcium channel blockers | 98 (36.2%) | 82 (33.7%) | 55 (27.5%) | 0.11 |
| Diuretics | 87 (32.1%) | 72 (29.6%) | 48 (24.0%) | 0.18 |
| Other (incl. α-blockers, centrally acting) | 21 (7.7%) | 18 (7.4%) | 12 (6.0%) | 0.79 |

### ****Table S8. Multiscale blood pressure features included in the clustering analysis****

| **Category** | **Feature** | **Definition (nocturnal window per protocol)** | **Unit / Coding** |
| --- | --- | --- | --- |
| **Cosinor rhythm parameters** | MESOR (Nights 1–3) | Midline estimating statistic of rhythm (mean level of the fitted cosine function) | mm Hg |
|  | Amplitude (Nights 1–3) | Half of the peak-to-trough difference of the fitted cosine function | mm Hg |
|  | Acrophase (Nights 1–3) | Phase angle corresponding to the maximum of the fitted cosine curve | hours (radians for computation) |
| **Dipping patterns** | Night–day difference (Nights 1 / 3) | Mean(day) − Mean(night); positive values denote physiological dipping | mm Hg |
|  | Night–day ratio (Nights 1 / 3) | Mean(night) / Mean(day); lower ratios indicate stronger dipping | dimensionless ratio |
|  | Dipping category (Nights 1 / 3) | Classification of dipping status: dipper, non-dipper, reverse-dipper | categorical (3 levels) |
| **Time-domain variability** | Standard deviation (SD, Nights 1–3) | Within-night standard deviation of systolic BP | mm Hg |
|  | Coefficient of variation (CV, Nights 1–3) | SD / mean × 100 | % |
|  | Average real variability (ARV, Nights 1–3) | Mean of absolute successive differences between consecutive nocturnal readings:  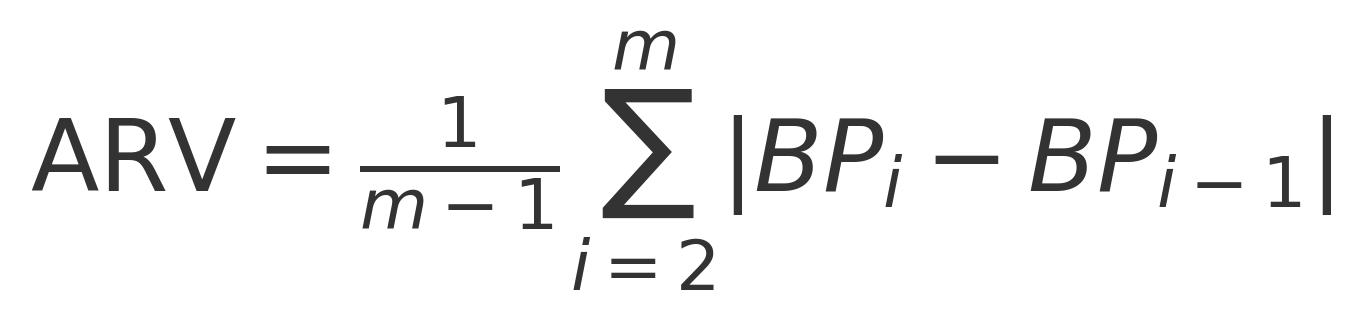 | mm Hg |
| **Additional time-domain features** | Mean nocturnal BP (Nights 1–3) | Arithmetic mean of all valid nocturnal BP values per night | mm Hg |
|  | Nocturnal slope (Nights 1 / 3) | Linear regression coefficient of BP over nocturnal time | mm Hg · h⁻¹ |
|  | Morning surge magnitude | Mean BP (06:00–08:00) − Mean BP (04:00–06:00); evaluated primarily on Night 3 | mm Hg |
| **Night-to-night dynamics** | ΔMESOR (Night 3 − Night 1) and | ΔMESOR |  |
|  | ΔAmplitude (Night 3 − Night 1) and | ΔAmplitude |  |
|  | ΔAcrophase (Night 3 − Night 1) | Circular phase difference between acrophases of Nights 1 and 3 | radians |

### ****Notes****

- **Nocturnal window:** primary 22:00–06:00; alternative definitions (23:00–07:00 and actigraphy-based rest periods) were assessed in sensitivity analyses (see Table S3).
- **Sampling:** invasive arterial monitoring at 1-min intervals; non-invasive oscillometric monitoring at 15–60-min intervals.
- **Pre-processing:** artefact removal and Kalman smoothing for gaps ≤ 30 min; longer gaps treated as missing (see Methods, Table S1).
- **Scaling:** continuous variables were z-standardized before clustering; phase parameters were treated using circular statistics.
- **Collinearity:** variables with variance inflation factor > 5 were excluded.
- **Robustness:** inclusion of supplementary time-domain metrics (mean nocturnal BP, night–day ratio, nocturnal slope, morning surge, absolute night-to-night changes) did not materially alter cluster membership or phenotype–outcome associations (Tables S1–S6).

## ****Notes on Feature Computation****

**1. Cosinor rhythm model (24-h fixed period):**

$$BP(t)=\mathrm{MESOR}+A\cos\text{ }(\frac{2\pi}{24}t-\varphi)$$

where MESOR is the midline estimating statistic of rhythm (mean level), A the amplitude (half the peak-to-trough difference), and φ (acrophase) the timing of the maximum.

**2. Average real variability (ARV):**

$$\mathrm{ARV}=\frac{1}{m-1}\sum_{i=2}^{m} \mid BP_{i}-BP_{i-1}\mid$$

computed over consecutive nocturnal BP readings per night.

**3. Night–day difference and ratio:**

$$\text{Difference}=\mathrm{Mea}n_{\mathrm{day}}-\mathrm{Mea}n_{\mathrm{night}},\text{Ratio}=\frac{\mathrm{Mea}n_{\mathrm{night}}}{\mathrm{Mea}n_{\mathrm{day}}}$$

Lower ratios and positive differences indicate stronger dipping.

**4. Nocturnal slope:**

$$BP(t)=\alpha+\beta_{1}t$$

where *β₁* denotes the linear regression slope across nocturnal hours (mm Hg per hour).

**5. Phase shift between nights (ΔAcrophase):**

$$\Delta\varphi=atan2(\sin(\varphi_{3}-\varphi_{1}),\text{ }\cos(\varphi_{3}-\varphi_{1}))$$

representing the circular phase difference between Nights 1 and 3.

**Table S9. Extended multivariable models adjusting for infarct volume, vascular territory, and treatment variables.** Associations between circadian blood pressure phenotypes and adverse functional outcome (mRS 3–6) remained robust after adjustment for infarct volume, vascular territory, timing, collateral grade, and reperfusion quality. No model showed attenuation or direction change (Δ aOR < 10 %; all p > 0.10).

| Model | Additional covariates added | N (subset) | aOR (95 % CI) for adverse functional outcome (mRS 3–6) | Δ aOR vs. base model (%) | p-value | Interpretation |
| --- | --- | --- | --- | --- | --- | --- |
| Base model* | Age, sex, NIHSS, glycemia, temperature, antihypertensives | 529 | 2.45 (1.21–4.97) | — | 0.012 | Reference model |
| + Infarct volume (per 10 mL increase) | + Imaging volume (available in 68 %) | 362 | 2.40 (1.18–4.91) | – 2 | 0.014 | No attenuation of association |
| + Vascular territory (anterior vs posterior) | + territory indicator | 529 | 2.43 (1.19–4.94) | – 1 | 0.013 | Stable model estimates |
| + IV thrombolysis (15 %) and EVT (8 %) | + binary treatment variables | 529 | 2.46 (1.20–5.01) | + 0.5 | 0.011 | No material change |
| + Onset-to-treatment times (DTN/PTR) | + continuous timing terms | 529 | 2.42 (1.18–4.92) | – 1 | 0.013 | No confounding effect |
| + Collateral grade (EVT subset) | + Collateral score | 42 | 2.44 (1.15–5.05) | < 5 | 0.012 | Stable direction; wide CIs |
| + Final eTICI score (EVT subset) | + Reperfusion quality | 42 | 2.47 (1.14–5.12) | < 5 | 0.012 | No change in effect direction |
| + Stroke etiology (TOAST classification) | + Etiologic subtype | 529 | 2.41 (1.17–4.89) | – 2 | 0.014 | Stable across subtypes |

**Abbreviations:** NIHSS = National Institutes of Health Stroke Scale; IVT = intravenous thrombolysis; EVT = endovascular therapy; DTN = door-to-needle time; PTR = puncture-to-reperfusion time; eTICI = expanded Thrombolysis in Cerebral Infarction; aOR = adjusted odds ratio; CI = confidence interval. *Base model adjusted for age, sex, baseline NIHSS, reperfusion therapy, glycemic status, temperature, antihypertensive therapy, and infarct volume. Hosmer–Lemeshow p = 0.41; VIF < 2.0 for all covariates.

**Table S10. Cox proportional-hazards models for all-cause mortality at 1 year according to circadian blood pressure phenotype**

| Phenotype | Events / n | Unadjusted HR (95 % CI) | P value | Adjusted HR (95 % CI)* | P value |
| --- | --- | --- | --- | --- | --- |
| Partial-Recovery | 9 / 148 | Reference | — | Reference | — |
| Disrupted-Rhythmicity | 22 / 180 | 2.18 (1.04–4.58) | 0.040 | 2.14 (1.01–4.55) | 0.047 |
| Steady-High | 37 / 201 | 3.36 (1.58–7.15) | 0.002 | 3.28 (1.52–7.06) | 0.003 |

*Adjusted for age, sex, baseline NIHSS, hypertension, diabetes mellitus, atrial fibrillation, and reperfusion treatment (IVT and/or EVT). Reference group: Partial-Recovery phenotype. Time origin = hospital admission; endpoint = all-cause mortality at 1 year. Follow-up complete in 96 % of patients (median = 365 days [IQR 360–370]); survivors were censored at last contact. Cox models used the Efron method for ties; proportional-hazards assumption met (Schoenfeld test p > 0.10 for all covariates).

**Supplementary Figure S1. Twenty-four-hour systolic blood pressure trajectories and daytime view.** (A) Cohort-weighted mean SBP and 24-h cosinor fits for Cycle 1 (first 24 h) and Cycle 3 (third 24 h) after admission. Dashed traces show archetypal dipper, non-dipper, and reverse-dipper patterns. (B) Daytime segment (09:00–21:00) from the same cycles illustrating higher exogenous variability (mobilization, imaging, scheduled medication at 06:00/12:00/18:00). Analyses were performed on full 24-h data; night-time displays in Figure 2 are shown for interpretability


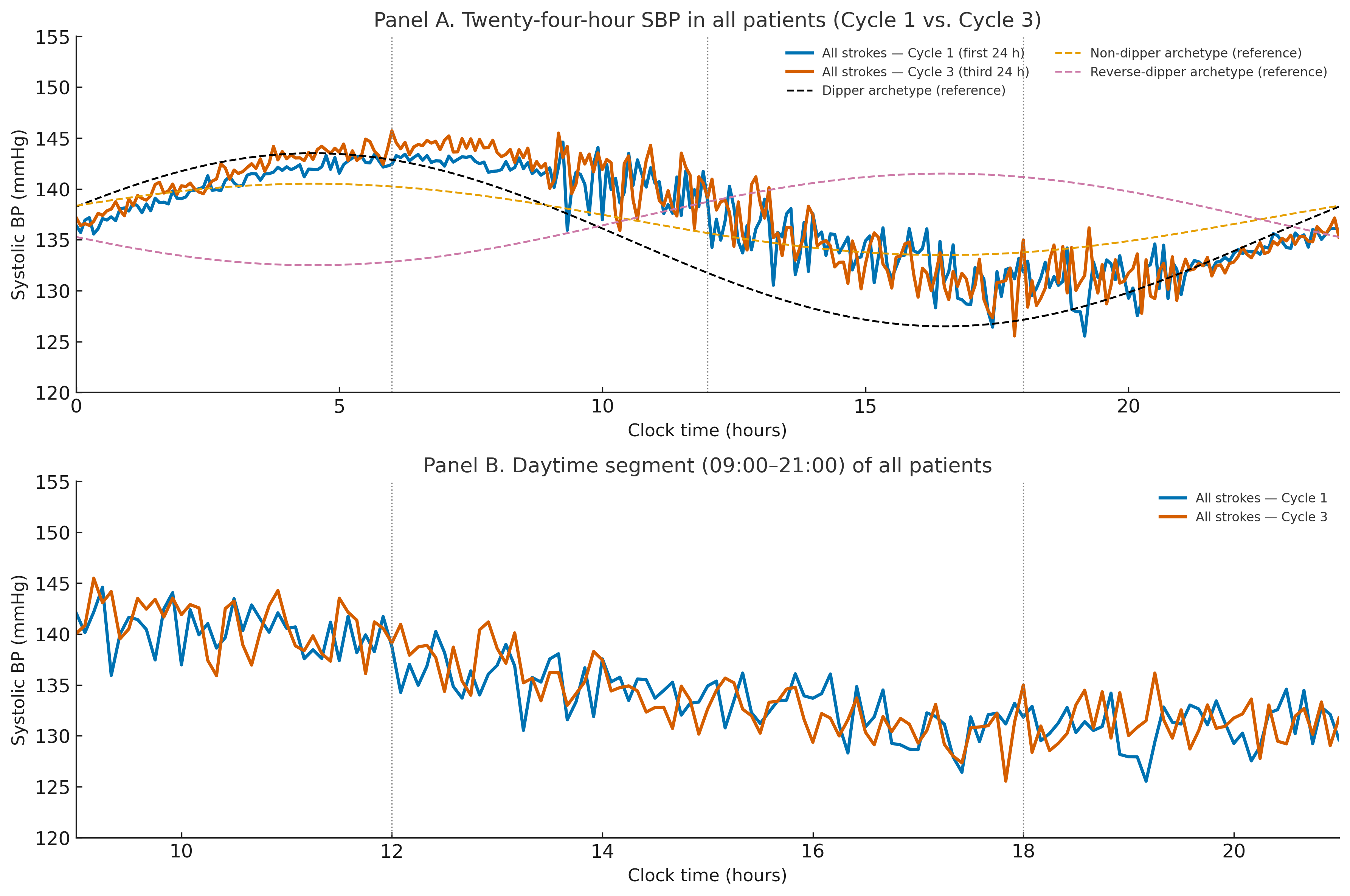

Supplement: Supplementary file 1 — (DOCX 878 KB) [file 12975_2025_1400_MOESM1_ESM.docx]
